# Supplementary material for: Understanding the needs of family caregivers of stroke patients with disabilities: A phenomenological study using the timing it right theory
Source: PLoS One. 2025 Jun 11;20(6):e0325671. doi: 10.1371/journal.pone.0325671 (PMC12157060; doi:10.1371/journal.pone.0325671)
Supplement: Attachment — Submitted filename: Response to Reviewers.docx [file pone.0325671.s002.docx]

**PONE-D-25-04561**

**The Needs of the Family Caregivers of Stroke Patients with Disabilities Based on Timing It Right Theory: A Phenomenological Qualitative Study**

**PLOS ONE**

Dear Editor-in-Chief,

Thank you very much for your careful review and constructive suggestions with regard to our manuscript **“****The Needs of the Family Caregivers of Stroke Patients with Disabilities Based on Timing It Right Theory: A Phenomenological Qualitative Study”.**

We have carefully evaluated all the comments and suggestions, and responded to them in our revised manuscript accordingly. All the authors agreed upon and approved the contents of the manuscript.Again, we appreciate the scholarly work by the Editors/Reviewers, and hope our detailed revisions have addressed all the criticisms raised by the reviewers.

Please contact us with any further questions and we are looking forward to your positive consideration.

Sincerely,

Fangqun Cheng

Department of Nursing

Xiangtan Central Hospital

Xiangtan, 411100, China

[445935584@qq.com](mailto:445935584@qq.com)

Please include the following items when submitting your revised manuscript:

A rebuttal letter that responds to each point raised by the academic editor and reviewer(s). You should upload this letter as a separate file labeled 'Response to Reviewers'.

**Answer:** Thank you for your suggestion.

We have included a separate **"Response to Reviewers"** document addressing each point raised.

A marked-up copy of your manuscript that highlights changes made to the original version. You should upload this as a separate file labeled 'Revised Manuscript with Track Changes'.

**Answer:** Thank you for your reminder.

We have provided a marked-up copy of the manuscript labeled **'****Revised Manuscript with Track Changes'** for your review.

An unmarked version of your revised paper without tracked changes. You should upload this as a separate file labeled 'Manuscript'.

**Answer:** Thank you for your reminder.

We have provided an unmarked version of the revised paper labeled **'Manuscript'** for your review.

If you would like to make changes to your financial disclosure, please include your updated statement in your cover letter. Guidelines for resubmitting your figure files are available below the reviewer comments at the end of this letter.

**Answer:** Thank you for your notice.

We do not need to make any changes to our financial disclosure. In addition, we have followed the guidelines provided for resubmitting the figure files.

If applicable, we recommend that you deposit your laboratory protocols in protocols.io to enhance the reproducibility of your results. Protocols.io assigns your protocol its own identifier (DOI) so that it can be cited independently in the future. For instructions see: https://journals.plos.org/plosone/s/submission-guidelines#loc-laboratory-protocols. Additionally, PLOS ONE offers an option for publishing peer-reviewed Lab Protocol articles, which describe protocols hosted on protocols.io. Read more information on sharing protocols at <https://plos.org/protocols?utm_medium=editorial-email&utm_source=authorletters&utm_campaign=protocols.>

**Answer:** Thank you for the thoughtful suggestion.

Our team carefully considered your recommendation. However, as we have provided a detailed description of our methods in the manuscript, we are not planning to deposit our laboratory protocols in protocols.io at this time. Nonetheless, we sincerely appreciate the suggestion and will keep it in mind for future projects.

Journal Requirements:

1. When submitting your revision, we need you to address these additional requirements.

Please ensure that your manuscript meets PLOS ONE's style requirements, including those for file naming. The PLOS ONE style templates can be found at

[https://journals.plos.org/plosone/s/file?id=wjVg/PLOSOne_formatting_sample_main_body.pdf](https://track.editorialmanager.com/CL0/https:%2F%2Fjournals.plos.org%2Fplosone%2Fs%2Ffile%3Fid=wjVg%2FPLOSOne_formatting_sample_main_body.pdf/1/010f0193d7653da7-5af1b3be-ca04-4e86-83c9-af444a5e9237-000000/zDm59Oi3QjjnKrReHcf_ySLoi6Z-m9amXOBphWA1KCI=189) and

[https://journals.plos.org/plosone/s/file?id=ba62/PLOSOne_formatting_sample_title_authors_affiliations.pdf](https://track.editorialmanager.com/CL0/https:%2F%2Fjournals.plos.org%2Fplosone%2Fs%2Ffile%3Fid=ba62%2FPLOSOne_formatting_sample_title_authors_affiliations.pdf/1/010f0193d7653da7-5af1b3be-ca04-4e86-83c9-af444a5e9237-000000/lv18rRxiXGip4bRyG2goQ-knIqF-T8cyySB73zzhPJ4=189)

**Answer:** Thank you for the suggestion.

We have carefully reviewed the PLOS ONE's style requirements and ensured that both our manuscript and file names fully comply with the provided guidelines.

2.Please state what role the funders took in the study.  If the funders had no role, please state: ""The funders had no role in study design, data collection and analysis, decision to publish, or preparation of the manuscript.""

If this statement is not correct you must amend it as needed.

Please include this amended Role of Funder statement in your cover letter; we will change the online submission form on your behalf.

**Answer:** Thank you for your reminder.

We have clarified in the cover letter that the funders provided support for the material printing cost during the questionnaire collection and the layout cost during the later publication of the article but had no role in study design, data analysis, decision to publish, or preparation of the manuscript.

3. We note that you have indicated that there are restrictions to data sharing for this study. For studies involving human research participant data or other sensitive data, we encourage authors to share de-identified or anonymized data. However, when data cannot be publicly shared for ethical reasons, we allow authors to make their data sets available upon request. For information on unacceptable data access restrictions, please see <http://journals.plos.org/plosone/s/data-availability#loc-unacceptable-data-access-restrictions.>

Before we proceed with your manuscript, please address the following prompts:

a) If there are ethical or legal restrictions on sharing a de-identified data set, please explain them in detail (e.g., data contain potentially identifying or sensitive patient information, data are owned by a third-party organization, etc.) and who has imposed them (e.g., a Research Ethics Committee or Institutional Review Board, etc.). Please also provide contact information for a data access committee, ethics committee, or other institutional body to which data requests may be sent.

b) If there are no restrictions, please upload the minimal anonymized data set necessary to replicate your study findings to a stable, public repository and provide us with the relevant URLs, DOIs, or accession numbers. Please see <http://www.bmj.com/content/340/bmj.c181.long> for guidelines on how to de-identify and prepare clinical data for publication. For a list of recommended repositories, please see <https://journals.plos.org/plosone/s/recommended-repositories.> You also have the option of uploading the data as Supporting Information files, but we would recommend depositing data directly to a data repository if possible

Please update your Data Availability statement in the submission form accordingly.

**Answer:** Thank you for your reminder.

In accordance with the informed consent agreements, the full transcripts of the interviews cannot be made publicly available due to privacy concerns. However, a summary of the thematic analysis, along with illustrative quotes that have been anonymized to protect participant confidentiality, are included in the manuscript. Requests for further data access may be considered on a case-by-case basis, subject to ethical review board approval and a data sharing agreement.

The datasets generated and/or analyzed during the current study are not publicly available due to ethical restrictions imposed by the Ethics Committee of Xiangtan Central Hospital. The data contain potentially identifying and sensitive patient information pertaining to mental health diagnoses and treatment histories, and the Research Ethics Committee deemed that public sharing, even in a de-identified format, could compromise patient privacy. Requests for data access should be directed to the Ethics Committee of Xiangtan Central Hospital by +86 731 58214988.

[Note: HTML markup is below. Please do not edit.]

Reviewers' comments:

Reviewer's Responses to Questions

**Comments to the Author**

1. Is the manuscript technically sound, and do the data support the conclusions?

The manuscript must describe a technically sound piece of scientific research with data that supports the conclusions. Experiments must have been conducted rigorously, with appropriate controls, replication, and sample sizes. The conclusions must be drawn appropriately based on the data presented.

Reviewer #1: Yes

**Answer:** Thank you for your positive feedback.

Reviewer #2: Partly

**Answer:** Thank you for your careful review.

We have made comprehensive revisions based on the editor's and reviewers' comments to ensure that experiments are conducted rigorously. Additionally, our team has rechecked and analyzed the data once more and consulted a statistical expert to verify the accuracy of our data analysis methods.

2. Has the statistical analysis been performed appropriately and rigorously?

Reviewer #1: Yes

**Answer:** Thank you for your positive feedback.

Reviewer #2: N/A

**Answer:** Thank you for your feedback.

3. Have the authors made all data underlying the findings in their manuscript fully available?

The [PLOS Data policy](http://track.editorialmanager.com/CL0/http://www.plosone.org/static/policies.action%23sharing/1/010f0195703f03ee-ecb6ac44-703e-45b9-aa5b-186dc79a1c05-000000/6bF5t2Qu3k6BqR8zhT4OsQnBZFuLy5Hpuk1VCUJXRV4=201) requires authors to make all data underlying the findings described in their manuscript fully available without restriction, with rare exception (please refer to the Data Availability Statement in the manuscript PDF file). The data should be provided as part of the manuscript or its supporting information, or deposited to a public repository. For example, in addition to summary statistics, the data points behind means, medians and variance measures should be available. If there are restrictions on publicly sharing data—e.g. participant privacy or use of data from a third party—those must be specified.

Reviewer #1: No

**Answer:** Thank you for raising this important point.

The data supporting this study (transcripts of qualitative interviews) cannot be publicly shared due to ethical restrictions protecting participant privacy. Our research protocol and informed consent documents explicitly stated that raw interview data would remain confidential and accessible only to the research team, as approved by the Ethics Committee of Xiangtan Central Hospital (register NO.2023-09-013).

Reviewer #2: No

**Answer:** Thank you for raising this important point.

The data supporting this study (transcripts of qualitative interviews) cannot be publicly shared due to ethical restrictions protecting participant privacy. Our research protocol and informed consent documents explicitly stated that raw interview data would remain confidential and accessible only to the research team, as approved by the Ethics Committee of Xiangtan Central Hospital (register NO.2023-09-013).

4. Is the manuscript presented in an intelligible fashion and written in standard English?

PLOS ONE does not copyedit accepted manuscripts, so the language in submitted articles must be clear, correct, and unambiguous. Any typographical or grammatical errors should be corrected at revision, so please note any specific errors here.

Reviewer #1: Yes

**Answer:** Thank you for your positive feedback.

Reviewer #2: No

**Answer:** Thank you for highlighting this important point.

We have made revisions based on your feedback and sought the assistance of a professional service for language editing to improve clarity and correctness. We appreciate your guidance and welcome any further concerns you may have.

5.Review Comments to the Author

Please use the space provided to explain your answers to the questions above. You may also include additional comments for the author, including concerns about dual publication, research ethics, or publication ethics. (Please upload your review as an attachment if it exceeds 20,000 characters)

**Answer:** Thank you for your thorough review and comments.

We have carefully considered your feedback and made the necessary revisions to address the issues raised. We sincerely appreciate your attention to detail and guidance in improving the manuscript. If you have any further concerns or additional comments, please do not hesitate to let us know.

Reviewer #1: I have read the manuscript, and it presents an interesting and valuable study. The writing is clear and provides meaningful insights into the target group.

**Answer:** Thank you for your positive assessment of our work.

We sincerely appreciate your positive feedback and recognition of the significance and clarity of our work. Your acknowledgment of the study's value and its contribution to understanding the target population is highly encouraging. We have carefully addressed all comments from the reviewers to further strengthen the manuscript. Thank you for your time and constructive evaluation.

While the title effectively reflects the study's scope, it lacks scientific precision. I suggest rephrasing it as 'Understanding the Needs of Family Caregivers of Stroke Patients with Disabilities: A Phenomenological Study Using the Timing It Right Theory' to enhance clarity and conciseness."

**Answer:** Thank you for your constructive suggestion.

We have revised the title as recommended to better align with the study’s theoretical framework and methodological approach. This modification enhances both clarity and scientific rigor, as noted in your feedback.

The abstract:It is well written, providing a clear and structured summary of the study.

**Answer:** Thank you for your kind acknowledgment of the abstract’s clarity and structure.

We aimed to succinctly convey the study’s purpose and key findings, and we are pleased this resonated with you.

Concerns:

• The Timing It Right (TIR) theory is introduced but could benefit from a very brief mention of its core premise in a phrase to guide unfamiliar readers.

**Answer:**Thank you for this valuable suggestion.

We have integrated a concise definition of the TIR theory into the revised abstract:"...based on the framework of the 'Timing It Right' (TIR) theory, a conceptual model emphasizing dynamic support aligned with caregivers' evolving needs across distinct care phases."This addition enhances clarity and contextualizes the theoretical foundation of the study.

• Check the grammar eg: "Caregivers’ experiences and needs were explored across five stages of care: diagnosis, stabilization, discharge preparation, adjustment, and adaptation." – suggested revision: "This study explored caregivers' experiences and needs across five key stages of care: diagnosis, stabilization, discharge preparation, adjustment, and adaptation."

**Answer:**We appreciate your meticulous attention to detail.

The sentence has been revised as suggested to improve grammatical structure and readability.All similar instances in the manuscript have been checked and revised to ensure consistency.

• The conclusion could be more assertive in emphasizing the study's contributions.

**Answer:** Thank you for this suggestion.

We have expanded the conclusion to emphasize practical contributions:"Practical implications include integrating multidisciplinary teams, leveraging telemedicine for continuity of care, and designing caregiver education programs aligned with Confucian family values."This addition clarifies how the findings can inform clinical practice and policy development.

Introduction:

• The problem statement currently focuses on the burden of caregiving but could be more explicit in defining the core issue (the need) that necessitates this research.

**Answer:** Thank you for your constructive suggestion.

We have revised the problem statement to explicitly define the core issue as the dynamic, phase-specific needs of caregivers. We have removed some contents related to the burden of care. And we have added some information about the needs of caregivers in the first and second paragraphs. These revisions have focused the core issue of the research on the needs of caregivers.

• The introduction mentions previous studies on caregivers' psychological burden, coping strategies, and social support. However, the specific gaps in existing literature should be more explicitly stated.

**Answer:** Thank you for your feedback.

We have explicitly highlighted gaps in prior research, including the static/fragmented perspectives and the lack of attention to cultural mediation (e.g., Confucian norms) and systemic challenges (e.g., urban-rural disparities). These additions are integrated into the fourth paragraph of the Introduction.

Methodology and tool:

• The study mentions member checking (participants reviewing transcripts), but triangulation (e.g., multiple researchers analyzing the data) should be clarified.

**Answer:** Thank you for your recommendation.

We clarified triangulation by specifying dual independent coding by researchers and third-party arbitration for discrepancies. This is detailed in the "Data Analysis" section under the revised Methods.

• It states that researchers had no prior relationships with participants, which is good. However, a reflexivity statement (acknowledging potential biases) would enhance transparency.

**Answer:** Thank you for your constructive suggestion.

 We have added a reflexivity statement in the "Rigor and Reflexivity" section to explicitly acknowledge potential biases arising from the research team’s professional roles (e.g., nursing administrators prioritizing systemic interventions versus clinical nurses focusing on practical challenges). This statement also outlines mitigation strategies, such as regular reflective discussions and peer debriefing, to ensure balanced interpretations. The revision enhances transparency by addressing how researcher perspectives were critically examined, aligning with your recommendation.

• The description of the study setting and participants' demographics helps, but a clearer discussion on how findings may apply beyond this sample would be beneficial.

**Answer:**Thank you for your constructive suggestion.

We have revised the "Participants and Sampling" section to explicitly discuss the potential applicability of findings beyond the study sample.These additions appear in the revised manuscript under the "Participants and Sampling" subsection, addressing how the study’s design and sample diversity support broader applicability. We stated that the diversity of sample enhances the potential applicability of findings to caregivers in similar socioeconomic and cultural contexts within China.

The results:

• The five caregiving stages (diagnosis, stabilization, discharge preparation, adjustment, adaptation) are well structured. Each stage is linked to a major theme, reflecting the evolving needs of caregivers.The subthemes highlight specific concerns, such as knowledge acquisition, rehabilitation skills, safety management, and emotional burden. Well done!

**Answer:**Thank you for your recognition of the thematic framework design.

The stages were carefully structured to reflect caregivers’ dynamic needs, and your positive feedback reinforces the validity of this approach.

Discussion:

• Deepen interpretation of findings by explaining why they emerged.

**Answer:** Thank you for your constructive suggestion.

In the discussion section, we elaborated on the findings of each stage and provided explanations to enhance the overall understanding and significance of the research. Additionally, according to your suggestion, we compared our findings with previous studies to identify similarities and discrepancies, which helped in validating our conclusions and suggesting potential areas for future exploration.

• Engage more critically with previous studies—highlighting what is new or different.

**Answer:** Thank you for your feedback.

We contrasted findings with prior studies, emphasizing novel themes like hospital navigation challenges and reliance on informal rehabilitation methods in China. For instance, the revised Discussion highlights how caregivers’ use of traditional massage diverges from structured programs in high-resource settings. These comparisons are integrated throughout the Thematic Analysis subsections.

• Provide clearer, stage-specific recommendations for practice and policy.

**Answer:** Thank you for your notice.

We refined recommendations to align with each care phase. Examples include:Diagnosis Phase: Augmented reality navigation systems for hospital logistics.Adaptation Phase: Policy reforms for subsidized home safety audits.These targeted strategies are detailed in the Conclusion and respective phase subsections.

• Expand the limitations section to address biases and generalizability.

**Answer:** Thank you for your notice.

We deepened the analysis of potential biases (e.g., purposive sampling favoring high-burden caregivers) and proposed combining stratified purposive sampling with quantitative validation to enhance representativeness. This addition strengthens the Limitations discussion.

• Strengthen the conclusion by emphasizing the study’s unique contributions.

**Answer:** Thank you for your recommendation.

We underscored the study’s strategies hold practical value across diverse healthcare settings. These contributions are explicitly stated in the Conclusion subsection.

Reviewer #2:

Abstract:

Specify the cultural context in the objective statement.

**Answer:**Thank you for your feedback.

The Objective statement has been revised to highlight the cultural context:"This study aims to explore the comprehensive care needs of family caregivers of stroke patients with disabilities in the Chinese cultural context, based on the framework..."This modification underscores the study’s focus on cultural influences unique to China.

Briefly mention cultural influences in the results.

**Answer:** Thank you for your suggestion.

We have explicitly highlighted cultural influences on caregivers' needs in the Results:

"Caregivers' needs were influenced by cultural expectations (e.g., filial piety norms requiring family-based caregiving), resource limitations, and their evolving role over time."This addition clarifies how Confucian cultural values directly shaped caregivers' responsibilities and demands, such as prioritizing family-led care over external support services.

Add a sentence on practical implications.

**Answer:** Thank you for this suggestion.

We have expanded the Conclusion section to emphasize practical implications:"Practical implications include integrating multidisciplinary teams, leveraging telemedicine for continuity of care, and designing caregiver education programs aligned with Confucian family values."This addition clarifies how the findings can inform clinical practice and policy development.

Introduction:

Streamline statistical data.

**Answer:** Thank you for your suggestion.

We removed redundant global cost data (e.g., $89.1 billion GDP impact) and emphasized rising stroke incidence trends ("The absolute number of incident strokes globally increased by 70.0% over three decades"). This revision appears in the first paragraph of the Introduction.

Introduce TIR theory earlier.

**Answer:** Thank you for your input.

We introduced the TIR theory at an appropriate position in the second paragraph in advance, improving logical coherence.

Improve transition between stroke burden and caregiver focus.

**Answer:** Thank you for your suggestion.

A restructured logic chain now connects epidemiological data to caregiver challenges: "imposing significant caregiving burdens..." transitions from economic impacts to familial roles, while "disproportionately bear" highlights inequitable burden distribution (Introduction, para 1). The subsequent TIR theory section bridges disease progression and dynamic caregiver needs, establishing a theoretical scaffold for the study’s focus.

Methods:

Clarify participant recruitment and sample size rationale.

**Answer:** Thank you for your feedback.

We clarified recruitment by specifying thematic saturation as the stopping criterion and maximum variation sampling to ensure diversity. This is included in the revised "Participants and Sampling" section.

Provide details on theme identification and validation.

**Answer:** Thank you for your feedback.

We have elaborated the details of theme validation steps, including peer debriefing and member checking, in the "Data Analysis" section. The six-phase analytical process is explicitly outlined.

Discuss reflexivity and researcher influence.

**Answer:** Thank you for your input.

We discussed how professional roles (e.g., nursing administrators vs. clinical nurses) might influence interpretations and mitigated this through regular reflective discussions and peer debriefing. This is addressed in the "Rigor and Reflexivity" section.

Results:

Present themes more concisely.

**Answer:** Thank you for your constructive suggestion.

We have streamlined the thematic titles and descriptions to enhance clarity. Redundant explanations in theme narratives were removed, focusing on core caregiver challenges. These revisions mainly appeared in the diagnosis phase and stabilization phase under "Thematic Analysis" subsections.

Add demographic context for quotes.

**Answer:** Thank you for your feedback.

We have appended demographic details to all participant quotes, including age, gender, occupation, and caregiving role. For example:"When he vomit, I don’t know what to do..." (A3, elderly female patient's wife)These additions contextualize quotes within caregivers’ lived experiences and are integrated throughout the "Thematic Analysis" subsections.

Reference and describe Fig. 1 in detail.

**Answer:** Thank you for your observation.

We expanded the description of Fig. 1 to clarify its role in first paragraph of the "Thematic Analysis" subsections. Added: "This framework highlights how caregivers' roles evolve in response to patient recovery trajectories and systemic constraints, transitioning from acute crisis management to chronic adaptation."

Discussion:

Critically address study limitations (e.g., single-site bias, lack of longitudinal data).

**Answer:** Thank you for your constructive suggestion.

We have critically addressed methodological constraints in both the Discussion and Limitations sections. In the concluding sentences of the Discussion's first paragraph, we explicitly state: "These interpretations should be contextualized by methodological limitations. The single-site design may underrepresent rural caregiving realities, while cross-sectional data limit tracking of need evolution. Future multi-center longitudinal studies could address these constraints." Additionally, the Limitations subsection now elaborates on strategies to mitigate single-site bias and proposes mixed-methods longitudinal designs for future validation, fully aligning with your recommendations.

Expand on policy and healthcare implications.

**Answer:** Thank you for your input.

We added policy-level recommendations, such as incentivizing hospitals to standardize follow-up protocols and training rural healthcare workers. The proposed implications are systematically integrated into the corresponding phase-specific subsections of the Discussion, where each care phase explicitly addresses how findings translate into actionable strategies.

Strengthen telemedicine discussion with specific studies/examples.

**Answer:** Thank you for your recommendation.

We incorporated specific examples, including a randomized controlled trial on home-based telerehabilitation demonstrating reduced caregiver burden (P < 0.001). This addition, along with references to systematic reviews, appears in the Adjustment Phase discussion.

Limitations:

Discuss the impact on generalizability.

**Answer:** Thank you for your constructive suggestion.

We expanded the discussion on generalizability by explicitly noting that findings may not fully apply to rural populations or regions with distinct healthcare infrastructures. This revision appears in the opening paragraph of the Limitations section.

Suggest ways to address limitations in future research.

**Answer:** Thank you for your feedback.

We added actionable recommendations for future studies, such as conducting multi-site research and employing longitudinal mixed-methods designs , to address sampling bias and contextual variability. These suggestions are integrated into the revised Limitations section.

Conclusion：

Specify practical implementation steps.

**Answer:** Thank you for your suggestion.

We specified phase-specific strategies under the revised Conclusion, aligning recommendations with distinct care phases.

Highlight broader societal impact.

**Answer:** Thank you for your input.

We emphasized the societal relevance of findings by linking interventions to resource-limited settings and aging populations, highlighting scalability for overwhelmed healthcare systems. This appears in the paragraph of the Conclusion.

General Comments：

Ensure consistency in terminology and formatting.

**Answer:** Thank you for your feedback.

We standardized terminology by replacing inconsistent terms like “period” and “stage” with “phase” throughout the manuscript. Besides, "diagnostic period" has been replaced by "diagnosis phase". These ensures coherence in framing the care continuum and aligns with the TIR theory’s phase-based framework.

Consider adding a demographic summary table.

**Answer:** Thank you for your suggestion.

We added a “Total (N=48) N(%)” column to Table 2 to summarize aggregate demographic data across all phases. This modification provides a comprehensive overview of participant characteristics (e.g., gender distribution, education levels, rural/urban residence) and enhances transparency in reporting sample diversity.

Deepen the discussion of study limitations.

**Answer:** Thank you for your notice.

We deepened the discussion of limitations by addressing selection bias and generalizability constraints. We proposed future strategies such as multi-site studies and longitudinal mixed-methods designs to mitigate these issues. These additions strengthen the critical reflection on methodological boundaries.
